# Does enhanced antibiotic de-escalation really have no benefit in the ICU?

**DOI:** 10.1186/s13054-017-1845-4

**Published:** 2017-11-02

**Authors:** Shingo Ohki, Shinichiro Ohshimo, Nobuaki Shime

**Affiliations:** Department of Emergency and Critical Care Medicine, Graduate School of Biomedical & Health Sciences, Hiroshima University, 1-2-3 Kasumi, Minami-ku, Hiroshima, 734-8551 Japan

In a recent issue of *Critical Care*, we read with interest the article by Trupka et al. [1], who investigated the utility of an enhanced antimicrobial de-escalation (EAD) program in mechanically ventilated patients with suspected pneumonia. They found that the EAD program did not affect the rate of antibiotic de-escalation or the duration of antibiotic therapy in intensive care units (ICUs). We are very interested in this research because it includes important findings for the indication of antimicrobial stewardship in ICUs.

However, several issues potentially affecting the results need to be addressed. First, the definition of ventilator-associated pneumonia (VAP) is unclear. In the study, 35% of the patients were classified as having pathogen-negative pneumonia. Appropriate antibiotic de-escalation depends on the precise evaluation of targeted microorganisms. Invasive respiratory sampling and quantitative culture may improve antibiotic treatment protocols in patients with VAP. Giantsou et al. [2] demonstrated that VAP patients diagnosed by bronchoalveolar lavage (BAL) were more likely to achieve antibiotic de-escalation than those diagnosed by tracheal aspiration. Further, Raman et al. [3] showed that it was safe to discontinue antibiotic treatment in VAP patients following negative quantitative BAL culture results. Therefore, the sampling and culture methods used for respiratory microorganisms might confound the evaluation of the utility of the EAD program.

Second, the time from initial antibiotic therapy to de-escalation was not reported, although the total antibiotic days were similar between the groups. Antimicrobial stewardship programs can reduce the time to de-escalation, which may reduce antibiotic resistance and other antibiotic-related adverse events. Carratalà et al. [4] demonstrated that stewardship of the antibiotic treatment in patients with community-acquired pneumonia reduced the time to oral antibiotic conversion, as well as total antibiotic days. Therefore, the time to de-escalation could be an important surrogate for evaluating the usefulness of an EAD program.

Finally, the report lacked information regarding the initial antibiotic therapy regimen. A recent meta-analysis demonstrated that initial inappropriate antibiotic therapy increased the risk of mortality in a hospital setting [5]. Further, if the spectrum of the initial antibiotic was sufficiently specific and narrow, de-escalation was not always necessary, which might affect the rate of de-escalation. Therefore, the initial antibiotic therapy regimen could be associated with the main results of this study.

In conclusion, we believe that provision of additional data by the authors will help us better understand the utility of EAD programs in ICUs.

## Abbreviations

BAL: Bronchoalveolar lavage
EAD: Enhanced antimicrobial de-escalation
ICU: Intensive care unit
VAP: Ventilator-associated pneumonia.
