# Supplementary material for: Does enhanced antibiotic de-escalation really have no benefit in the ICU?
Source: Crit Care. 2017 Nov 2;21:270. doi: 10.1186/s13054-017-1845-4 (PMC5667459; doi:10.1186/s13054-017-1845-4)
Supplement: Related texts

# Reply: Author’s response

**Authors:** Marin H. Kollef

We thank Ohki and colleagues for their letter regarding our manuscript examining the addition of an enhanced antimicrobial de-escalation (EAD) program to a high-intensity daytime staffing model in an academic ICU [1, 6]. Unfortunately, the authors seemed to have failed to appreciate our conclusions. In no way does our study suggest that antibiotic de-escalation is not achievable in the ICU setting. In fact, among all patients eligible for antimicrobial de-escalation, it was achieved in two-thirds of them in both study groups. This is one of the highest numbers for de-escalation in this type of population reported in the medical literature. The EAD program simply did not increase this percentage in the intervention group. This is likely a reflection of the ICUs in which we carried out this project. All of the ICUs at Barnes-Jewish Hospital are closed units with high-intensity multidisciplinary staffing providing continuous patient care to include clinical pharmacists. There is also a long history of antimicrobial stewardship being practiced within these ICUs [7].

Ohki et al. suggest that the diagnostic approach for establishing the presence of pneumonia could have influenced our results. This is very unlikely given that we employ a robust methodology for achieving this which includes performing routine bronchoalveolar lavage in our intubated patients with suspected pneumonia, as well as utilizing quantitative bacterial cultures and molecular arrays to identify respiratory viruses. We also found no difference in the time to de-escalation of antibiotics between both study groups, which was approximately 2 days after the start of initial therapy. This is simply a function of the time it takes to perform antimicrobial susceptibility testing in our microbiology laboratory. Lastly, antibiotic therapy is protocolized in our ICUs and reviewed by the ICU team including the clinical pharmacists on a daily basis [7]. We care for a high acuity patient population with a high prevalence of antibiotic resistance [8]. It is very unlikely that the initial spectrum of empiric therapy influenced our results.
